# Altered anterior segment biometrics in cataract patients with retinitis pigmentosa: a propensity-matched analysis suggests patterns of zonular weakness

**DOI:** 10.7717/peerj.20760

**Published:** 2026-02-19

**Authors:** Yonglin Chen, Zhiheng Lian, Yongguo Xiang, Hong Cheng, Chong Tang, Yanlin Yang, Liang Liang, Juan Kang, Ke Hu, Shijie Zheng

**Affiliations:** 1 The First Affiliated Hospital of Chongqing Medical University, Chongqing Key Laboratory of Prevention and Treatment on Major Blinding Diseases, Chongqing Eye Institute, Chongqing Branch (Municipality Division) of National Clinical Research Center for Ocul, Chong Qing, China; 2 Changdu People’s Hospital of Xizang, Tibet, Qamdo, China

**Keywords:** Retinitis pigmentosa, Cataract, Zonule, Anterior segment biometrics

## Abstract

**Purpose:**

To explore the spatial structure of the anterior segment biometrics in cataract patients with retinitis pigmentosa (RP).

**Methods:**

This is a retrospective study. We conducted a propensity score matching analysis (1:3) on anterior segment data of RP patients and normal cataract patients who underwent cataract surgery from August 2023 to February 2025. Baseline variables (age, gender, axial length, and Pentacam Nucleus Staging (PNS) score) were balanced to compare the differences in anterior segment biometric measurements between the two groups.

**Results:**

We analyzed the anterior segment data of 37 cataract patients with RP and 102 simple cataract patients. The RP group exhibited a thicker lens (*p* < 0.001), a more anterior lens position (*p* = 0.003), a shallower anterior chamber depth (*p* < 0.001), and greater corneal astigmatism (*p* < 0.001). The shallower anterior chamber depth showed a positive correlation with a more anterior lens position (*r* = 0.84, *p* < 0.001) and the relative lens position (*r* = 0.59, *p* < 0.001).

**Discussions:**

RP patients exhibit a thicker lens, a more anteriorly positioned lens, shallower anterior chamber depth, and greater corneal astigmatism. These changes can be explained by zonular weakness. Decision-making surrounding intraocular lens selection in eyes with RP, cataract, and zonular compromise may be enhanced through proper identification of preoperative biometric irregularities in the anterior segment.

## Introduction

Retinitis pigmentosa (RP) is an inherited, bilateral degenerative condition characterized mainly by the gradual decline of rod and cone photoreceptors (Testa et al., 2014). Non-syndromic RP has a global prevalence of 1 in 4,000, impacting over 1 million individuals worldwide (Hamel, 2006). RP presents with night blindness, followed by early loss of peripheral visual field, which may ultimately result in loss of central vision (Hamel, 2006). In 20% to 30% of cases, RP presents alongside systemic conditions, a form referred to as RP (Verbakel et al., 2018). The occurrence of anterior segment diseases can complicate the process of RP. Cataract is one of the most common complications of RP (Tan et al., 2021). Posterior subcapsular cataract (PSC) is the most prevalent form of cataract, especially in individuals with Usher syndrome and autosomal recessive RP (Auffarth et al., 1997). The potential association between RP and glaucoma remains a subject of debate. The combination of RP and glaucoma may be coincidental because researchers have found no significant differences in the incidence of RP associated with glaucoma as compared with simple glaucoma, either in terms of prevalence or in terms of anterior segment parameters (Xu et al., 2017). However, several studies report a higher prevalence of both open-angle and closed-angle glaucoma in RP patients compared to the general population (Hung & Chen, 2022a; Hung & Chen, 2022b; Ko et al., 2014; Pradhan et al., 2022). Prior research has documented the presence of an intraocular inflammatory response in patients with RP (Bacherini et al., 2024; Yoshida et al., 2013a; Yoshida et al., 2013b). Studies show elevated levels of inflammatory mediators and cells in the anterior chamber of RP patients (Tao et al., 2024). Aqueous flashes are associated with disease progression in patients with RP (Fujiwara et al., 2020; Nishiguchi et al., 2019), and are also a risk factor for the development of posterior subcapsular cataracts (Fujiwara et al., 2017). This chronic inflammatory state contributes to a higher rate of post-cataract surgery complications in RP patients, such as capsular contraction and posterior capsular opacification, compared to the general population (Dikopf et al., 2013; Fujiwara et al., 2017).

To date, the anterior segment anatomy of RP patients has received limited research attention. However, other inherited ocular diseases may involve changes in anterior segment structures. For instance, researchers have found that Marfan syndrome is often associated with reduced corneal curvature and thinner central corneal thickness, and when accompanied by lens subluxation, it may also involve greater corneal astigmatism (Konradsen et al., 2012). We hypothesized that chronic intraocular inflammation in RP leads to alterations in anterior segment structures, which may influence the development and progression of other anterior segment complications. Therefore, this study aimed to use propensity score matching to rigorously compare anterior segment biometric parameters between RP and non-RP cataract patients, which would then isolate the specific effects of RP on anterior segment anatomy.

Anterior segment symmetry can be due to normal variations in iris-lens anatomy, as well as lens zonular compromise. Performing such assessments on patients with RP before cataract surgery may help predict the existence of subclinical zonular issues, which increase the risk of intraoperative events. Previous studies have been centered on non-quantitative descriptions; this paper aims to fill that research gap by evaluating biometric variation *via* propensity-matched analyses.

## Patients and Methods

This is a retrospective study. The data were obtained from patients who underwent cataract surgery and received comprehensive ophthalmologic examinations at the Department of Ophthalmology, the First Affiliated Hospital of Chongqing Medical University (anonymous review) between August 2023 and February 2025. The project was approved by the Ethics Committee of the First Affiliated Hospital of Chongqing Medical University. The study is compliant with the Declaration of Helsinki. This study has been granted an exemption from informed consent by the ethics committee. Patients aged 25 years and older were included in the study. We excluded cataract patients with the Pentacam Nucleus Grading System (PNS) lens turbidity greater than or equal to 4 to avoid the effect of severe cataract on anterior segment parameters. In addition, instances where data was incomplete were also omitted. In addition, patients were excluded from the primary analysis if they had a history of any of the following: (1) anterior segment diseases (*e.g.*, corneal opacity, uveitis); (2) ocular surgery (*e.g.*, cataract, glaucoma, or vitreoretinal surgery); (3) ocular trauma that could potentially alter anterior segment anatomy; or (4) systemic diseases known to affect the eye, such as Marfan syndrome. Monocular (right eye) data were obtained for each patient for analysis, and left eye data were included for analysis when the right eye did not meet the inclusion criteria. Participants were categorized into two groups: cataract with combined RP patients (RP group) and cataract without combined RP patients (NRP group).

## Ocular Biometric Assessment

All patients underwent a comprehensive ocular examination, including visual acuity assessment; intraocular pressure (IOP) (non-contact tonometer) measurement; slit lamp biomicroscopy and fundoscopy; Humphrey visual field testing; routine Optical Coherence Tomography (OCT) (fundus imaging), wide-angle fundus photography after pupil dilation, IOL Master 700, and Pentacam. PNS is an objective method of evaluating the degree of cataract as determined by Scheimpflug imaging on a scale of 0–5, with higher scores indicating greater cataract severity. The Topcon Corneal Endothelial Cytometer measures central corneal thickness (CCT) and corneal endothelial cell density (ECD). Electroretinography was performed to clarify the diagnosis of RP in patients with fundus photography and night blindness symptoms suggestive of RP. The IOL Master 700 (Carl Zeiss AG, Oberkochen, Germany) is the first scanning-source optical coherence tomography (OCT)-based biometry device that provides anatomical detail in complete longitudinal slices of the eye. All patients underwent IOL Master 700 biometry before pupil dilation. Records of all measurements were extracted, including axial length (AL), corneal curvature (K), anterior chamber depth (ACD), lens thickness (LT), corneal diameter (WTW), and corneal astigmatism (Ast). The ACD is the distance between the tear film and the anterior capsule of the lens. The lens position (LP), posterior lens surface position (PPL), and relative lens position (RLP) were then calculated by the following equations: LP is the distance from the corneal apex to the central plane of the lens; PPL is the distance from the corneal apex to the posterior surface of the lens; and RLP is the ratio of LP to AL.

\begin{eqnarray*}\mathrm{LP}=\mathrm{ACD}+1/2\mathrm{LT} \end{eqnarray*}

\begin{eqnarray*}\mathrm{PPL}=\mathrm{ACD}+\mathrm{LT} \end{eqnarray*}

\begin{eqnarray*}\mathrm{RLP}=\mathrm{LP}/\mathrm{AL}. \end{eqnarray*}

The diagnosis of RP is based on the following characteristics: (1) symptoms of night blindness; (2) dilated fundus photography showing bone spicule-like pigment deposits originating from the peripheral retina, retinal vascular attenuation, waxy pallor of the optic disc, and varying degrees of retinal atrophy; and (3) electroretinography (ERG) demonstrating a greater reduction in the amplitude of A-waves and B-waves in the scotopic system (rods) compared to the photopic system (cones).

## statistical Analysis

Propensity score matching was executed employing R version 4.2.2 (R Project for Statistical Computing, Vienna, Austria). A 1:3 ratio propensity score matching was employed to control and reduce the imbalance and impact of selection bias between the RP group and the NRP group. Variables suspected to be associated with the spatial structure of the anterior segment in the literature and knowledge domain were considered potential confounding factors. The following identified confounders were used to calculate the propensity score: suspected with the spatial structure of the immediate node in the knowledge domain, gender, age, axial length, and cataract extent (PNS score). Propensity scores were calculated using logistic regression analysis. The caliper width was 0.05. Covariate balance post-propensity matching was assessed by comparing standardized mean differences for each baseline variable, ensuring values were below 0.1, thereby confirming sufficient balance between the RP and non-RP groups.

Data were analyzed using SPSS software version 27.0. We compared demographic and ocular biometric parameters between the RP group and the NRP group, including age, sex, AL, PNS, ACD, LT, RLT, CCT, ECD, WTW, K, and Ast. Continuous variables were expressed as M ± SD, and categorical variables were expressed as percentages. The Shapiro–Wilk W test quantitative variables for normality. If the quantitative variables follow a normal distribution, they were tested using independent samples t-tests; otherwise, the Mann–Whitney U-test was used. Correlation analysis was performed using the Spearman correlation coefficient. The interpretation of the correlation coefficient (r) was adopted from established guidelines: 0.00–0.10, negligible; 0.10–0.39, weak; 0.40–0.69, moderate; 0.70–0.89, strong; and 0.90–1.00, very strong. For all analyses, statistical significance was set at a two-tailed *p*-value of <0.05.

*Post-hoc* power analyses, conducted using PASS (v.2021) based on the observed effect sizes (Cohen’s d), demonstrated exceptionally high statistical power for the key outcomes. Given the actual sample size (RP group *n* = 37, control group *n* = 102) and an alpha of 0.05, achieved power was > 0.99 for ACD >0.99 for LT, and >0.99 for LP.

## Results

**Figure 1:**
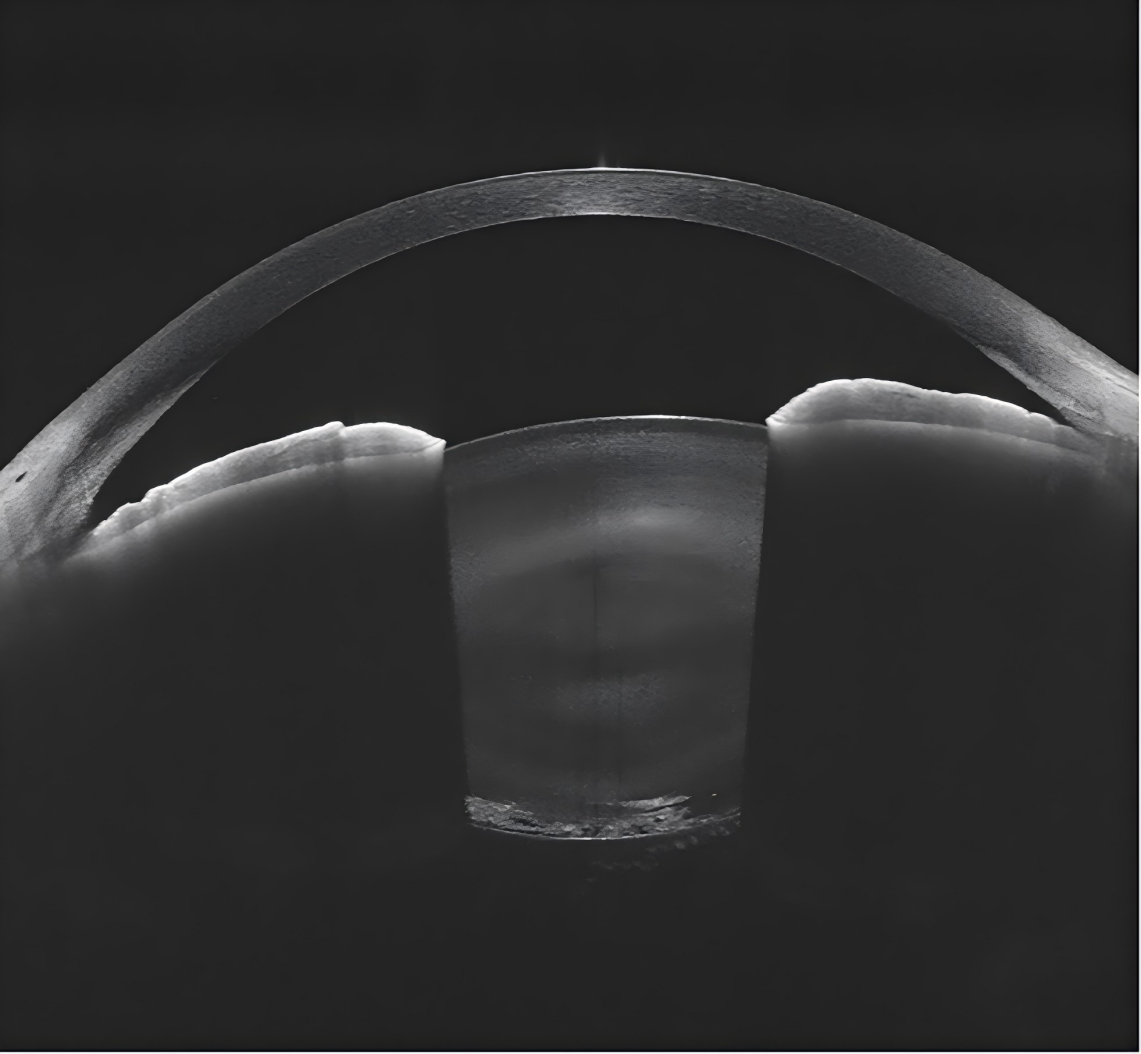
Anterior segment OCT of RP patient (posterior subcapsular cataract). This OCT image reveals the development of posterior subcapsular opacities in the lens of a retinitis pigmentosa patient.

This study enrolled 39 Chinese cataract patients with RP and 243 Chinese cataract patients without RP. We used propensity score matching (1:3) to control differences in age, sex, axial length, and cataract severity (PNS score) between the RP group and the non-RP (NRP) group. Ultimately, 37 cataract patients with RP and 102 cataract patients without RP were successfully matched (Table 1). Among the RP patients, the most common type of cataract was posterior subcapsular cataract (Fig. 1). There were no statistically significant differences between the two groups in age, sex, axial length, PNS score, K, CCT, ECD, WTW, or PPL (*P* > 0.05). Compared to the NRP group, the RP group exhibited greater corneal astigmatism (*P* < 0.001), thinner lens thickness (*P* < 0.001), a more anteriorly positioned lens (*P* = 0.003), and relative lens position (*P* = 0.031) (Table 2). These differences collectively imply that RP may correlate with a series of biometric alterations indicative of zonular compromise, even in the absence of overt subluxation.

**Table 1:** Demographic properties of the groups.

	RP group	NRP group	*p*
n	37	102	
Age (y)	55.38 ± 10.53	54.13 ± 10.06	0.637
Sex (male)	0.51	0.45	0.514
PNS	0.65 ± 0.63	0.67 ± 0.75	0.873
AL (mm)	23.69 ± 1.49	23.80 ± 1.47	0.703

**Notes.**

*p*-value of sex from chi-square test and the other *p*-values are from Mann–Whitney U-tests

PNS Pentacam Nucleus Staging AL axial length

**Figure 2:**
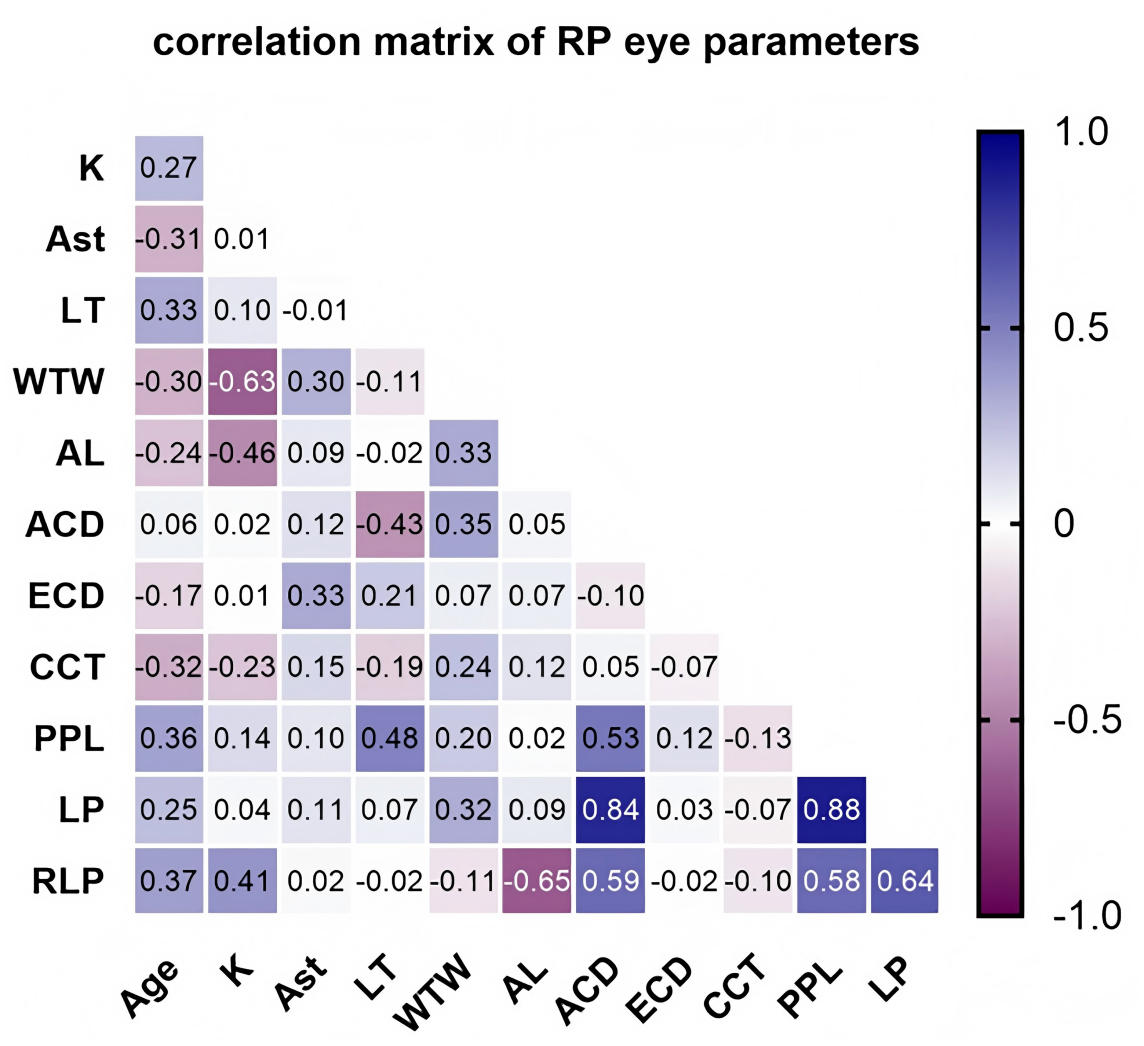
Correlation matrix of RP eye parameters. The numbers represent Spearman’s correlation coefficients, with darker colors indicating stronger correlations. Darker shades of blue indicate stronger positive correlations, while darker shades of purple represent stronger negative correlations.

In the correlation analysis, anterior segment measurement parameters, as well as age and AL, were included (Fig. 2). In the correlation analysis of ACD, ACD showed no significant correlation with age (*r* = 0.06, *P* = 0.738) or AL (*r* = 0.05, *P* = 0.791). It exhibited a weak negative correlation with lens thickness (LT) (*r* =−0.43, *P* = 0.008), a weak positive correlation with white-to-white distance (WTW) (*r* = 0.35, *P* = 0.034), a strong positive correlation with lens position (LP) *r* = 0.84, *P* < 0.001) and a moderate positive correlation with relative lens position (RLP) (*r* = 0.59, *P* < 0.001).In the correlation analysis between Age and lens parameters, Age was weakly positively correlated with LT (*r* = 0.33, *P* = 0.064) and LP (*r* = 0.37, *P* = 0.024), and not significantly correlated with RLP (*r* = 0.25, *P* = 0.133).In the correlation analysis between AL and lens parameters, AL had no significant correlation with LT (*r* = −0.02, *P* = 0.522) and LP (*r* = 0.09, *P* = 0.700), and a moderate positive correlation with RLP (*r* =−0.65, *P* < 0.001). In the correlation analysis of ECD, there was no significant correlation between ECD and Age (*r* = 0.06, *P* = 0.302), AL (*r* = 0.07, *P* = 0.686), and CCT (*r* =−0.07, *P* = 0.686). The robust correlation between lens position and anterior chamber depth substantiates the hypothesis that zonular weakness affects anterior chamber configuration.

**Table 2:** Ocular properties of the groups.

	RP group	NRP group	*p*
n	37	102	
K (D)	44.31 ± 1.70 (43.74, 44.87)	44.36 ± 1.46 (44.08, 44.65)	0.493
Ast (D)	1.13 ± 0.84 (0.89, 1.37)	0.84 ± 0.49 (0.75, 0.94)	0.029
CCT (μm)	519.84 ± 32.24 (509.09, 530.59)	531.85 ± 34.68 (525.04, 538.67)	0.068
ECD	2,895.51 ± 478.16 (2,736.09, 3,052.94)	2,827.83 ± 382.28 (2,752.75, 2,902.92)	0.073
ACD (mm)	2.79 ± 0.36 (2.67, 2.91)	3.29 ± 0.37 (3.22, 3.36)	<0.001
WTW (mm)	11.70 ± 0.41 (11.56, 11.84)	11.71 ± 0.41 (11.63, 11.79)	0.702
LT (mm)	4.79 ± 0.38 (4.67, 4.92)	4.19 ± 0.40 (4.12, 4.27)	<0.001
LP (mm)	5.18 ± 0.34 (5.03, 5.30)	5.39 ± 0.26 (5.33, 5.44)	0.003
PPL (mm)	7.58 ± 0.42 (7.44, 7.32)	7.48 ± 0.29 (7.42, 7.54)	0.168
RLP	0.220 ± 0.018 (0.213, 0.226)	0.227 ± 0.012 (0.224, 0.229)	0.031

**Notes.**

Continuous variables are presented as mean ± standard deviation (95% confidence interval). *p*-values are from Mann–Whitney U-tests.

ACD anterior chamber depth K Corneal curvature Ast Astigmatism CCT Central corneal thickness ECD Endothelial cell density WTW White to white LT Lens thickness LP Lens position PPL Posterior surface position of the lens RLP Relative position of the lens

## Discussion

We conducted a comparative analysis of anterior segment parameters in cataract patients with and without RP, using propensity score matching (1:3) to control for confounders such as age, sex, axial length, and PNS score. The results showed that the RP group had a thicker LT, a more anteriorly positioned lens, a more posteriorly positioned lens posterior surface, a shallower ACD, and greater corneal astigmatism. Inflammatory processes such as free radical and cytokine release have been shown to precede degeneration of key components in the extracellular matrix of lens zonules. This degenerative mechanism may facilitate anterior lens displacement in RP, akin to biomechanical alterations characteristic of eyes with pseudo-exfoliation syndrome (Hayashi et al., 2024).

Zonular weakness is a frequent anterior segment complication in RP (Dikopf et al., 2013; Nguyen et al., 2023), which may be due to intraocular inflammation. We found that RP patients had a greater LT (.79 ± 0.38 mm) than reported for a large cohort which is 24,013, of typical cataract patients (4.51 ± 0.42 mm). In contrast, the LT of our normal control group (4.19 ± 0.40) was smaller than their reported result. This discrepancy may be due to our patient group being younger (54.13 ± 10.06 years) than the cohort in that study (62.5 ± 13.6 years) (Meng et al., 2021). Some researchers used anterior segment OCT to compare the lens morphology of cataract patients with and without RP. They found an increase in lens thickness and a decrease in the radius of curvature of the anterior lens surface, suggesting that these changes might be caused by zonular weakness (Iida et al., 2024). Sofija Andjelic et al. used scanning and transmission electron microscopy to examine the lenses of patients with RP and discovered abnormal structures in the lens epithelium, including holes, thinning, and degradation. These abnormalities may allow aqueous humor to flow into the lens, causing it to swell and thicken (Andjelic et al., 2017). In our results, there was no linear relationship between AL and LT, but LT was positively correlated with age. Previous researchers have also observed this phenomenon in normal eyes (Meng et al., 2021). In summary, our finding of increased lens thickness in RP patients may be linked to several factors, including underlying zonular weakness.

The prevalence of primary angle-closure glaucoma was higher in the Chinese population of RP patients (2.2%) than in normal subjects (Wang et al., 2022). Zonular weakness is common in RP patients, which may lead to anterior lens displacement and a shallower anterior chamber depth. In our results, the ACD in RP patients was significantly shallower than that in the control group and strongly correlated with anterior lens displacement. A shallower anterior chamber depth is a risk factor for angle-closure glaucoma (Yuan et al., 2024). Our findings suggest that the predisposition of RP patients to angle-closure glaucoma is likely not coincidental. This predisposition may be attributed to zonular weakness in RP, which can cause anterior lens displacement and a shallower anterior chamber, and these are effects potentially compounded by a thicker lens.

We found that the LP in RP patients showed no significant correlation with age. However, previous studies have reported that in normal patients, the lens tends to shift forward with increasing age (Namba et al., 2024). We found that the extent of lens displacement did not correlate significantly with patient age but was closely linked to the presence of anterior chamber flare in RP. Therefore, anterior lens displacement in RP appears to be more strongly associated with intraocular inflammation than with the aging process (Na et al., 2017).

In our statistical analysis, RP patients exhibited greater corneal astigmatism. Similarly, some researchers have observed greater corneal astigmatism in patients with Marfan syndrome, particularly in those with lens dislocation (Kinori et al., 2017; Konradsen et al., 2012). The mechanical forces transmitted by the zonules can deform the lens and may also alter the contour of the eyewall, indirectly influencing corneal shape (Bassnett, 2021). Supporting a link between zonular integrity and corneal shape, studies of Marfan syndrome have reported that the axis of lens dislocation correlates with the axis of corneal astigmatism (Maumenee, 1981). Previous studies have also found that RP patients exhibit thinning of the central corneal thickness and a reduction in corneal curvature under the influence of zonular weakness (Iida et al., 2024). We also noted thinner central corneas in RP patients compared to controls, although this difference was not statistically significant (*P* = 0.098), which may be related to the small sample size. In conclusion, the greater corneal astigmatism we observed in RP patients may be a clinical sign of underlying zonular weakness.

Anterior chamber inflammation may affect corneal endothelial cell density and morphology, as observed in patients with anterior uveitis (Alfawaz et al., 2016). Despite the documented chronic anterior chamber inflammation in RP (Yoshida et al., 2013a), we found no significant reduction in corneal endothelial cell density compared to controls, suggesting this low-grade inflammation does not critically compromise the endothelium. In normal individuals, thinner CCT may be associated with lower corneal endothelial cell density (Miao et al., 2024), but we did not observe such a correlation in RP patients. In conclusion, we found no evidence of reduced corneal endothelial cell density in RP patients.

This study is significant as it is the first to compare anterior segment parameters between Chinese RP patients and normal individuals. However, some limitations should be discussed. First, the sample size was limited, which may affect the statistical power and generalizability of our findings. Larger-scale studies are warranted. Second, we lacked direct assessment of zonular integrity (*e.g.*, *via* ultrasound biomicroscopy), relying instead on indirect biometric parameters. Third, the cross-sectional design precludes follow-up data, leaving the longitudinal changes in anterior segment anatomy unresolved. Finally, as the participants were recruited from tertiary referral centers, there is a potential for selection bias towards more advanced cases, which may not represent the broader RP population.

## Conclusion

RP patients exhibit a thicker lens, a more anteriorly positioned lens, shallower anterior chamber depth, and greater corneal astigmatism. These changes can be explained by zonular weakness. The findings of this study delineate unique anterior segment biometric characteristics that may suggest zonular weakness among patients with RP. Timely identification of these biometric changes may facilitate preoperative risk evaluation, surgical procedure adjustment, and intraocular lens fixation techniques in patients with RP.

## Supplemental Information

10.7717/peerj.20760/supp-1 Supplemental Information 1 Raw data prior to propensity score matching

10.7717/peerj.20760/supp-2 Supplemental Information 2 Raw data after propensity score matching

10.7717/peerj.20760/supp-3 Supplemental Information 3 Propensity Score Matching Analysis Code

10.7717/peerj.20760/supp-4 Supplemental Information 4 codebook

## Additional Information and Declarations

### Data Availability

The following information was supplied regarding data availability: The raw data are available in the Supplemental Files.

## References

[ref-1] Alfawaz AM, Holland GN, Yu F, Margolis MS, Giaconi JA, Aldave AJ (2016). Corneal endothelium in patients with anterior uveitis. Ophthalmology.

[ref-2] Andjelic S, Drašlar K, Hvala A, Hawlina M (2017). Anterior lens epithelium in cataract patients with retinitis pigmentosa - scanning and transmission electron microscopy study. Acta Ophthalmologica.

[ref-3] Auffarth GU, Tetz MR, Krastel H, Blankenagel A, Völcker HE (1997). Complicated cataracts in various forms of retinitis pigmentosa type and incidence. Ophthalmologe.

[ref-4] Bacherini D, Maggi L, Faraldi F, Sodi A, Vannozzi L, Mazzoni A, Capone M, Virgili G, Vicini G, Falsini B, Cosmi L, Viggiano P, Rizzo S, Annunziato F, Giansanti F, Liotta F (2024). CD3+CD4-CD8- double-negative lymphocytes are increased in the aqueous humor of patients with retinitis pigmentosa: their possible role in mediating inflammation. International Journal of Molecular Sciences.

[ref-5] Bassnett S (2021). Zinn’s zonule. Progress in Retina and Eye Research.

[ref-6] Dikopf MS, Chow CC, Mieler WF, Tu EY (2013). Cataract extraction outcomes and the prevalence of zonular insufficiency in retinitis pigmentosa. American Journal of Ophthalmology.

[ref-7] Fujiwara K, Ikeda Y, Murakami Y, Funatsu J, Nakatake S, Tachibana T, Yoshida N, Nakao S, Hisatomi T, Yoshida S, Yoshitomi T, Ishibashi T, Sonoda KH (2017). Risk factors for posterior subcapsular cataract in retinitis pigmentosa. Investigative Ophthalmology and Visual Science.

[ref-8] Fujiwara K, Ikeda Y, Murakami Y, Tachibana T, Funatsu J, Koyanagi Y, Nakatake S, Shimokawa S, Yoshida N, Nakao S, Hisatomi T, Ishibashi T, Sonoda KH (2020). Aqueous flare and progression of visual field loss in patients with retinitis pigmentosa. Investigative Ophthalmology and Visual Science.

[ref-9] Hamel C (2006). Retinitis pigmentosa. Orphanet Journal of Rare Diseases.

[ref-10] Hayashi K, Yoshida M, Manabe SI, Hirata A (2024). High-risk factors for zonular complications during cataract surgery in eyes with pseudoexfoliation syndrome. British Journal of Ophthalmology.

[ref-11] Hung MC, Chen YY (2022a). Association between retinitis pigmentosa and an increased risk of primary angle closure glaucoma: a population-based cohort study. PLOS ONE.

[ref-12] Hung MC, Chen YY (2022b). Patients with retinitis pigmentosa may have a higher risk of developing open-angle glaucoma. Journal of Ophthalmology.

[ref-13] Iida M, Masuda Y, Ohira R, Ichihara K, Komatsu K, Shiba T, Iwaki H, Oki K, Nakano T (2024). Ocular shape of cataract with retinitis pigmentosa: a case-control study. Journal of Cataract and Refractive Surgery.

[ref-14] Kinori M, Wehrli S, Kassem IS, Azar NF, Maumenee IH, Mets MB (2017). Biometry characteristics in adults and children with marfan syndrome: from the marfan eye consortium of Chicago. American Journal of Ophthalmology.

[ref-15] Ko YC, Liu CJ, Hwang DK, Chen TJ, Liu CJ (2014). Increased risk of acute angle closure in retinitis pigmentosa: a population-based case-control study. PLOS ONE.

[ref-16] Konradsen TR, Koivula A, Kugelberg M, Zetterström C (2012). Corneal curvature, pachymetry, and endothelial cell density in Marfan syndrome. Acta Ophthalmologica.

[ref-17] Maumenee IH (1981). The eye in the Marfan syndrome. Transactions of the American Ophthalmological Society.

[ref-18] Meng J, Wei L, He W, Qi J, Lu Y, Zhu X (2021). Lens thickness and associated ocular biometric factors among cataract patients in Shanghai. Eye and Vision.

[ref-19] Miao AO, Lin P, Qian D, Xu J, Lu YI, Zheng T (2024). Association between endothelial cell density and corneal thickness in medium, short, and long eyes of Han Chinese cataract patients. American Journal of Ophthalmology.

[ref-20] Na KH, Kim HJ, Kim KH, Han S, Kim P, Hann HJ, Ahn HS (2017). Prevalence, age at diagnosis, mortality, and cause of death in retinitis pigmentosa in Korea-A nationwide population-based study. American Journal of Ophthalmology.

[ref-21] Namba H, Maeda N, Tsukamoto M, Utsunomiya H, Kaneko Y, Nishitsuka K, Yamashita H, Ohta Y, Usui T, Sugimoto M (2024). Associations of ocular anterior segment structures with sex and age: the Yamagata study (Funagata). Japanese Journal of Ophthalmology.

[ref-22] Nguyen XT, Thiadens A, Fiocco M, Tan W, McKibbin M, Klaver CCW, Meester-Smoor MA, Van Cauwenbergh C, Strubbe I, Vergaro A, Pott JR, Hoyng CB, Leroy BP, Zemaitiene R, Khan KN, Boon CJF (2023). Outcome of cataract surgery in patients with retinitis pigmentosa. American Journal of Ophthalmology.

[ref-23] Nishiguchi KM, Yokoyama Y, Kunikata H, Abe T, Nakazawa T (2019). Correlation between aqueous flare and residual visual field area in retinitis pigmentosa. British Journal of Ophthalmology.

[ref-24] Pradhan ZS, Shroff S, Bansod A, Poornachandra B, Shetty A, Devi S, Rao DAS, Puttaiah NK, Rao HL (2022). Prevalence of primary angle-closure disease in retinitis pigmentosa. Indian Journal of Ophthalmology.

[ref-25] Tan L, Long Y, Li Z, Ying X, Ren J, Sun C, Meng X, Li S (2021). Ocular abnormalities in a large patient cohort with retinitis pigmentosa in Western China. BMC Ophthalmology.

[ref-26] Tao Y, Fukushima M, Shimokawa S, Zhao H, Okita A, Fujiwara K, Takeda A, Mukai S, Sonoda KH, Murakami Y (2024). Ocular and serum profiles of inflammatory molecules associated with retinitis pigmentosa. Translational Vision Science & Technology.

[ref-27] Testa F, Rossi S, Colucci R, Gallo B, Di Iorio V, Della Corte M, Azzolini C, Melillo P, Simonelli F (2014). Macular abnormalities in Italian patients with retinitis pigmentosa. British Journal of Ophthalmology.

[ref-28] Verbakel SK, Van Huet RAC, Boon CJF, Den Hollander AI, Collin RWJ, Klaver CCW, Hoyng CB, Roepman R, Klevering BJ (2018). Non-syndromic retinitis pigmentosa. Progress in Retina and Eye Research.

[ref-29] Wang DD, Gao FJ, Hu FY, Cao WJ, Xu P, Huang Y, Sun XH, Wu JH (2022). Clinical and genetic analysis of retinitis pigmentosa with primary angle closure glaucoma in the Chinese population. Current Eye Research.

[ref-30] Xu J, Ouyang Z, Yang Y, Cai X, Wang Z, Lin M, Zhang X, Liu X, Yu M (2017). Ocular biometry in primary angle-closure glaucoma associated with retinitis pigmentosa. JJournal of Ophthalmology.

[ref-31] Yoshida N, Ikeda Y, Notomi S, Ishikawa K, Murakami Y, Hisatomi T, Enaida H, Ishibashi T (2013a). Clinical evidence of sustained chronic inflammatory reaction in retinitis pigmentosa. Ophthalmology.

[ref-32] Yoshida N, Ikeda Y, Notomi S, Ishikawa K, Murakami Y, Hisatomi T, Enaida H, Ishibashi T (2013b). Laboratory evidence of sustained chronic inflammatory reaction in retinitis pigmentosa. Ophthalmology.

[ref-33] Yuan Y, Xiong R, Wang W, Xu BY, Liao C, Yang S, Li C, Zhang J, Yin Q, Zheng Y, Friedman DS, Foster PJ, He M (2024). Long-term risk and prediction of progression in primary angle closure suspect. JAMA Ophthalmology.

