# Supplementary material for: Altered anterior segment biometrics in cataract patients with retinitis pigmentosa: a propensity-matched analysis suggests patterns of zonular weakness
Source: PeerJ. 2026 Feb 19;14:e20760. doi: 10.7717/peerj.20760 (PMC12925417; doi:10.7717/peerj.20760)
Supplement: Supplemental Information 4 [file peerj-14-20760-s004.docx]

**We interpreted the categorical data as follows:**

group（0:cataract patients without RP; 1:cataract patients with RP）

sex（1:male; 2:female）
